# Supplementary material for: PRDX6 augments selenium utilization to limit iron toxicity and ferroptosis
Source: Nat Struct Mol Biol. 2024 Jun 12;31(8):1277–85. doi: 10.1038/s41594-024-01329-z (PMC11327102; doi:10.1038/s41594-024-01329-z)
Supplement: Supplementary file 1 — Supplementary Fig. 1 [file 41594_2024_1329_MOESM1_ESM.pdf]

nature structural & molecular biology

Supplementary information https://doi.org/10.1038/s41594-024-01329-z

PRDX6 augments selenium utilization to limit iron toxicity and ferroptosis

In the format provided by the authors and unedited

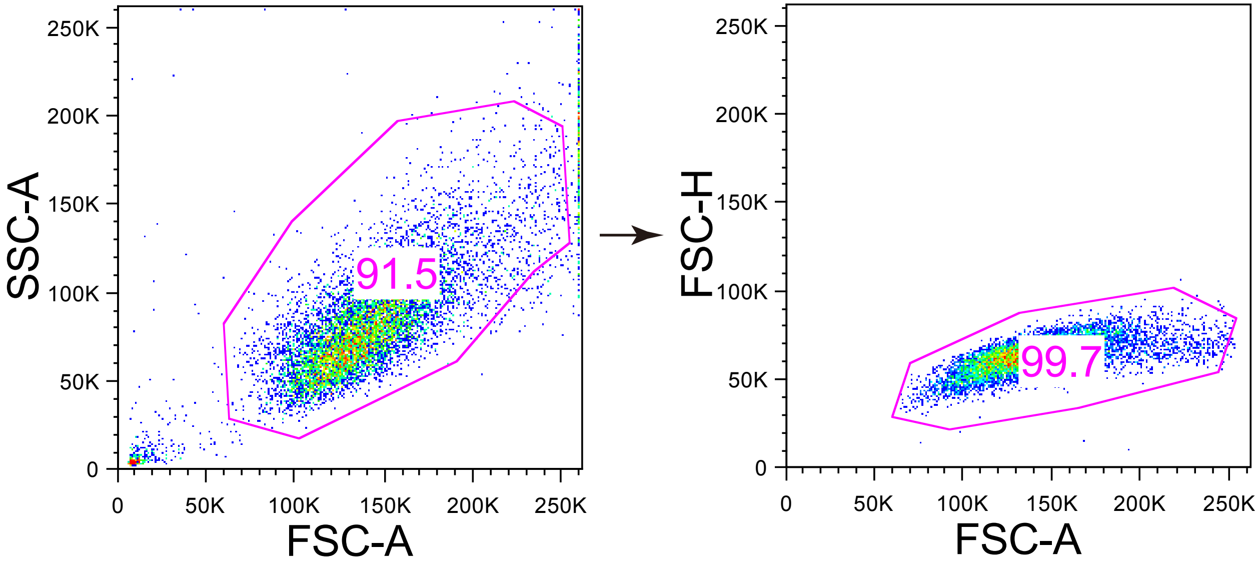

Supplementary Fig.1:An example for the gating strategy of flow cytometry
