# Supplementary material for: PRDX6 augments selenium utilization to limit iron toxicity and ferroptosis
Source: Nat Struct Mol Biol. 2024 Jun 12;31(8):1277–85. doi: 10.1038/s41594-024-01329-z (PMC11327102; doi:10.1038/s41594-024-01329-z)
Supplement: Supplementary file 2 — Reporting Summary [file 41594_2024_1329_MOESM2_ESM.pdf]

nature portfolio

Corresponding author(s): Hiroaki Fujita, Kazuhiro Iwai

Last updated by author(s): February 22, 2024

Reporting Summary

Nature Portfolio wishes to improve the reproducibility of the work that we publish. This form provides structure for consistency and transparency in reporting. For further information on Nature Portfolio policies, see our Editorial Policies and the Editorial Policy Checklist.

Statistics

For all statistical analyses, confirm that the following items are present in the figure legend, table legend, main text, or Methods section.

n/a	Confirmed	
☐	☒	The exact sample size (*n*) for each experimental group/condition, given as a discrete number and unit of measurement
☐	☒	A statement on whether measurements were taken from distinct samples or whether the same sample was measured repeatedly
☐	☒	The statistical test(s) used AND whether they are one- or two-sided<br>*Only common tests should be described solely by name; describe more complex techniques in the Methods section.*
☒	☐	A description of all covariates tested
☐	☒	A description of any assumptions or corrections, such as tests of normality and adjustment for multiple comparisons
☐	☒	A full description of the statistical parameters including central tendency (e.g. means) or other basic estimates (e.g. regression coefficient) AND variation (e.g. standard deviation) or associated estimates of uncertainty (e.g. confidence intervals)
☐	☒	For null hypothesis testing, the test statistic (e.g. *F*, *t*, *r*) with confidence intervals, effect sizes, degrees of freedom and *P* value noted<br>*Give P values as exact values whenever suitable.*
☒	☐	For Bayesian analysis, information on the choice of priors and Markov chain Monte Carlo settings
☒	☐	For hierarchical and complex designs, identification of the appropriate level for tests and full reporting of outcomes
☒	☐	Estimates of effect sizes (e.g. Cohen's *d*, Pearson's *r*), indicating how they were calculated

Our web collection on statistics for biologists contains articles on many of the points above.

Software and code

Policy information about availability of computer code

Data collection	Western blotting anaylysis was measured by a LAS4000mini or LAS3000 instrument (GE Healthcare).Cell viability assay was measured by SpectraMax M5(Molecular Device). Immunofluorescence images were collected using Fv1000 microscopy (Olympus). DNA and RNA concentrations were measured by Nanodrop2000 (Thermo).Protein concentration was measured by SpectraMax M5(molecular device) or V750 spectrophotometer (JASCO). GPX assay (absorbance at 340 nm) was measured by SpectraMax M5 (Molecular Devices).RT-qPCR data were obtained by ABI ViiA7 Real-Time PCR system (Applied Biosystems). Luciferase reporter was measured by a Lumat Luminometer (Berthold). The AMP product was measured by plate reader Nivo (PerkinElmer). Cell survival data on iCelligence system were acquired by using RTCA iCelligence Software (ACEA Biosciences). ICP-MS was done by Agilent 8800 ICP-MS/MS (Agilent Technologies). LC-ESI-Q-TOF analysis was performed using 6545XT AdvanceBio LC/Q-TOF (Agilent Technologies) connected to the Agilent HPLC system. Flow cytometry data was collected by using FACS Canto II (BD Biosciences) and FACS Diva software ver 6.1.2 (Becton Dickinson).
Data analysis	GraphPad Prism9 version 9.4.0 was used to perform statistical analysis. CRISPR screen data was analyzed by MAGeCK pipeline. Microsoft Exel ver16.72 was used for data analysis. Image Gauge ver4.22 (FUJIFILM) was used for chemiluminescent images. ViiA7 RUO Software ver1.2.3 (Thermo Fisher Scientific) was used for RNA expression analysis. Recording data of cell survival using iCelligence system were analyzed by RTCA Software Lite version2.2.1(ACEA Biosciences). Immunofluorescence images were analyzed by ImageJ (ver2.3.0). FlowJo software (Tomy Digital Biology, version 9.9.6) was used for all analyses of flow cytometry data. The modification analysis of the active center cysteine (Cys47) was performed using MassHunter BioConfirm software ver10.0 (Agilent Technologies). Figures in the manuscript were arranged and converted into PDF files by using illustrator version 27.4.1 (Adobe).

For manuscripts utilizing custom algorithms or software that are central to the research but not yet described in published literature, software must be made available to editors and reviewers. We strongly encourage code deposition in a community repository (e.g. GitHub). See the Nature Portfolio guidelines for submitting code & software for further information.

Data

Policy information about availability of data

All manuscripts must include a data availability statement. This statement should provide the following information, where applicable:

- Accession codes, unique identifiers, or web links for publicly available datasets
- A description of any restrictions on data availability
- For clinical datasets or third party data, please ensure that the statement adheres to our policy

Data of differential gene expression in tumor and normal tissues were obtained from TNMplot (https://tnmplot.com/analysis/). All data are available in the article and the supplementary information, and from the corresponding authors upon reasonable request.

Human research participants

Policy information about studies involving human research participants and Sex and Gender in Research.

Reporting on sex and gender	N/a
Population characteristics	N/a
Recruitment	N/a
Ethics oversight	N/a

Note that full information on the approval of the study protocol must also be provided in the manuscript.

Field-specific reporting

Please select the one below that is the best fit for your research. If you are not sure, read the appropriate sections before making your selection.

☒ Life sciences ☐ Behavioural & social sciences ☐ Ecological, evolutionary & environmental sciences

For a reference copy of the document with all sections, see nature.com/documents/nr-reporting-summary-flat.pdf

Life sciences study design

All studies must disclose on these points even when the disclosure is negative.

Sample size	No statistical tests were used to determine sample size. Sample size was determined on the previous studies in the field using similar experiment paradigms [PMID: 34031600 and 31634899], and to give sufficient values to conduct standard statistical tests.
Data exclusions	No data were excluded from the analyses.
Replication	All experiments were reproduced at least twice, each with similar results (the expression checks shown in extended data Figures 1g and 3a were single experiments).
Randomization	Not applicable as no animal studies were performed.
Blinding	Blinding was not done because the investigator needs to know the treatment groups in order to perform the experiments.

Reporting for specific materials, systems and methods

We require information from authors about some types of materials, experimental systems and methods used in many studies. Here, indicate whether each material, system or method listed is relevant to your study. If you are not sure if a list item applies to your research, read the appropriate section before selecting a response.

Materials & experimental systems

n/a	Involved in the study
☐	☒ Antibodies
☐	☒ Eukaryotic cell lines
☒	☐ Palaeontology and archaeology
☒	☐ Animals and other organisms
☒	☐ Clinical data
☒	☐ Dual use research of concern

Methods

n/a	Involved in the study
☒	☐ ChIP-seq
☐	☒ Flow cytometry
☒	☐ MRI-based neuroimaging

Antibodies

Antibodies used

anti-PRDX6 (Proteintech, 13585-1-AP; western blotting [WB], 1:2,000; Proximity ligation assay [PLA], 1:100), anti-SEPHS2 (Proteintech, 14109-1-AP; WB, 1:2,000), anti-GPX4 (Proteintech, 67763-1-AP; WB, 1:2,000), anti-FSP1 (Proteintech, 20886; WB, 1:2000), anti-SCLY (Proteintech, 67606-1-Ig; WB, 1:2000), anti-GPX4 (Santa Cruz, sc-166570; WB, 1:2,000), anti-SELN (Santa Cruz, sc-365824; WB, 1:2,000), anti-GPX1/2 (Santa Cruz, sc-133160; WB, 1:2,000), anti-PSTK (Santa Cruz, sc-373991; WB, 1:2,000), anti-FBXL5 (Santa Cruz, sc-390102; WB, 1:2,000), anti-FTH1 (Santa Cruz, sc-376594; WB, 1:300), anti-ACSL4 (Santa Cruz, sc-365230; WB, 1:2000), anti-PDSS2 (Santa Cruz, sc-515137; WB, 1:2000), anti-ferritin (Sigma-Aldrich, F6136; WB, 1:2,000), anti-IRP2 (in-house; WB, 1:1000), anti-LRP8 (abcam, ab108208; WB, 1:2000), anti-b-Actin (Sigma-Aldrich, A5316; WB, 1:15,000), anti-Tubulin (CEDARLANE, CLT9002; WB, 1:5,000), anti-Myc (Merck, 05-724; WB, 1:2,000; PLA, 1:200), anti-HA (MBL, M180-3; WB, 1:2000), anti-Ubiquitin K48-specific (Merck, ZRB2150; WB, 1:2000), anti-p62 (Wako, 018-22141; WB, 1:2,000), HRP-linked antimouse IgG (Cell Signaling, #7076; WB, 1:10,000), and HRP-linked antirabbit IgG (GE Healthcare, NA934; WB, 1:10,000).

Validation

Primary antibodies were validated in this study or used in previous articles for WB of mouse or human sample.

Anti-PRDX6 (Proteintech, 13585-1-AP), anti-SEPHS2 (Proteintech, 14109-1-AP), Anti-GPX4 (Proteintech, 67763-1-AP) and (Santa Cruz, sc-166570), anti-GPX1/2 (Santa Cruz, sc-133160), anti-PSTK (Santa Cruz, sc-373991), anti-FBXL5 (Santa Cruz, sc-390102), anti-ACSL4 (Santa Cruz, sc-365230), anti-PDSS2 (Santa Cruz, sc-515137), have been validated by knockout of the endogenous genes with CRISPR/Cas9.
Anti-FTH1 (Santa Cruz, sc-376594), anti-ferritin (Sigma-Aldrich, F6136) and Anti-IRP2 (in-house) have been validated in previous publication (PMID: 35318808)
Anti-FSP1 (Proteintech, 20886) has been validated in previous publication (PMID: 31634900).
Anti-SELN (Santa Cruz, sc-365824) and anti-LRP8 (abcam, ab108208) have been validated in previous publication (PMID: 35637349).

Validation detail for the other commercial antibodies is on the manufacture's website that is attached as following.
Anti-SCLY (Proteintech, 67606-1-Ig);https://www.ptglab.com/products/SCLY-Antibody-67606-1-Ig.htm; and has been also validated by expression of tagged-SCLY construct.
Anti-b-Actin (Sigma-Aldrich, A5316); https://www.sigmaaldrich.com/JP/en/product/sigma/a5316
Anti-Tubulin (CEDARLANE, CLT9002); https://www.cedarlanelabs.com/Products/Detail/CLT9002
Anti-Myc (Merck, 05-724); https://www.merckmillipore.com/JP/en/product/Anti-Myc-Tag-Antibody-clone-4A6,MM_NF-05-724
Anti-HA (MBL, M180-3); https://ruo.mbl.co.jp/bio/e/dtl/A/?pcd=M180-3
Anti-Ubiquitin K48-specific (Merck, ZRB2150); https://www.sigmaaldrich.com/JP/en/product/sigma/zrb2150
Anti-p62 (Wako, 018-22141); https://labchem-wako.fujifilm.com/us/product/detail/W01W0101-2214.html
HRP-linked antimouse IgG (Cell Signaling, #7076); https://www.cellsignal.com/products/secondary-antibodies/anti-mouse-igg-hrp-linked-antibody/7076?country=JP&language=en
HRP-linked antirabbit IgG (GE Healthcare, NA934); https://www.cytivalifesciences.com/en/us/shop/protein-analysis/blotting-and-detection/blotting-standards-and-reagents/amersham-ecl-hrp-conjugated-antibodies-p-06260

Eukaryotic cell lines

Policy information about cell lines and Sex and Gender in Research

Cell line source(s)

Mouse embryonic fibroblasts (MEFs) were generated in-house. HepG2 was gifted by Dr. Koichi Nakajima (Osaka City University), originally purchased from ATCC (HepG2 : HB-8065). HEK293T was gifted by Dr. Eijiro Nakamura (Kyoto University), originally purchased from RIKRN RBC (293T : RCB2202). PLATE was gifted by Dr. Toshio Kitamura (Tokyo University). A549, H226, H460 and H1975 were gifted by Dr. Atsuyasu Sato (Kyoto University), originally purchased from ATCC (A549 : CCL-185, H226 : CRL-5826, H460 : HTB-177, H1975 : CRL-5908). SK-N-DZ and HeLa cells were purchased from ATCC (SK-N-DZ : CRL-2149, Hela : CCL-2). PANC-1 and MIA Paca-2 cells were purchased from RIKEN RBC (PANC-1 : RCB2095, MIA Paca-2 : RCB2094). NB-1 cells were purchased from JCRB (NB-1 : JCRB0621).

Authentication

None of the cell lines used were not authenticated.

Mycoplasma contamination

All cell lines tested negative for mycoplasma contamination.

Commonly misidentified lines
(See ICLAC register)

No commonly misidentified cell line was used.

Flow Cytometry

Plots

Confirm that:

☒ The axis labels state the marker and fluorochrome used (e.g. CD4-FITC).

☒ The axis scales are clearly visible. Include numbers along axes only for bottom left plot of group (a 'group' is an analysis of identical markers).

☒ All plots are contour plots with outliers or pseudocolor plots.

☒ A numerical value for number of cells or percentage (with statistics) is provided.

Methodology

Sample preparation	Cells were plated in a 6-well plate and treated with FAC. After 1.5–24 h, the medium was removed, and the cells were labeled with DMEM containing 10 uM BODIPY 581/591 C11 at 37°C for 30 min. Cells were then washed three times with PBS and detached from the plate using trypsin, and green fluorescence was measured by flow cytometry using a BD FACSCanto II cytometer
Instrument	FACSCanto II (BD Biosciences) for flow cytometric analyses
Software	FACS Diva software ver 6.1.2 (Becton Dickinson) for data collection. FlowJo version 9.9.6 for data analysis.
Cell population abundance	At least 5000 cells were analyzed for each sample.
Gating strategy	FSC-area vs SSC-area was used to gate for the bulk populations of cells. Initial cell population gating (FSC-Area vs FSC-height) was adopted to make sure doublet exclusion and only single cell was used for analysis.

☒ Tick this box to confirm that a figure exemplifying the gating strategy is provided in the Supplementary Information.
