# Supplementary figures and images for: PRDX6 augments selenium utilization to limit iron toxicity and ferroptosis
Source: Nat Struct Mol Biol. 2024 Jun 12;31(8):1277–85. doi: 10.1038/s41594-024-01329-z (PMC11327102; doi:10.1038/s41594-024-01329-z)

Fig. 1a

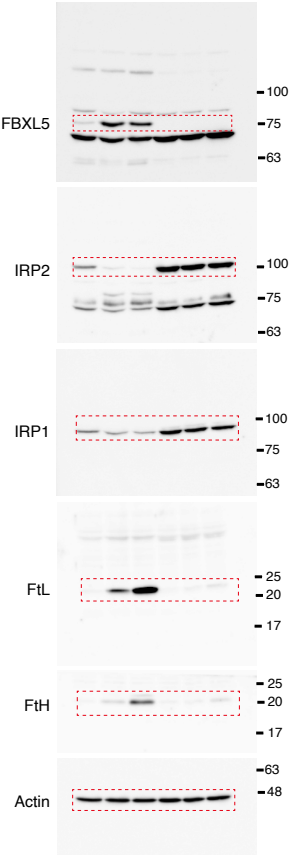

Supplement: Supplementary file 4 — Uncropped western blots [file 41594_2024_1329_MOESM4_ESM.pdf]

Fig. 2a

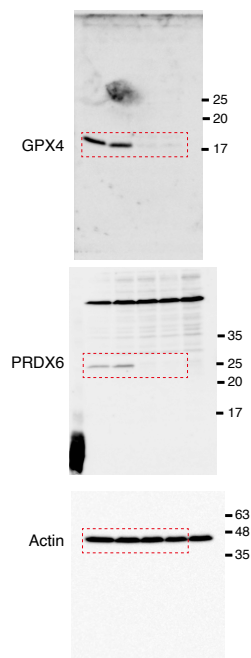

Fig. 2f

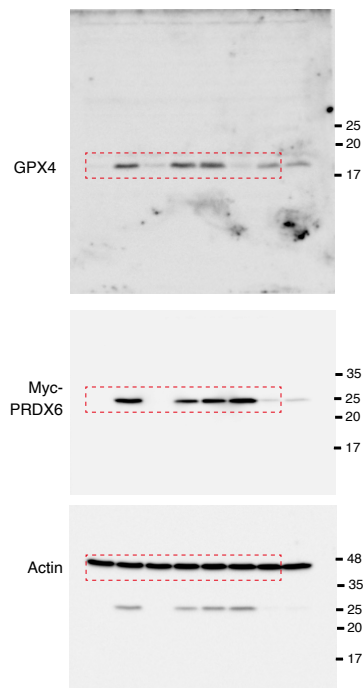

Fig. 2h

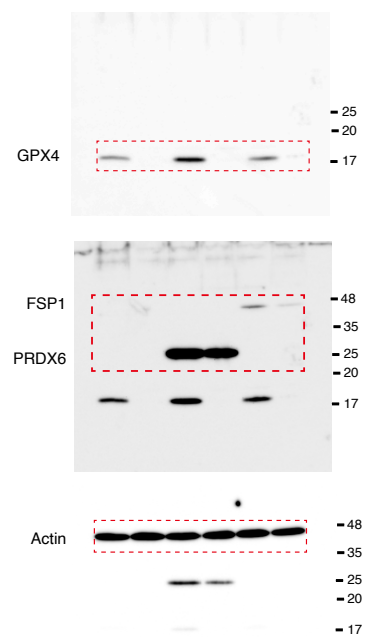

Supplement: Supplementary file 5 — Uncropped western blots [file 41594_2024_1329_MOESM5_ESM.pdf]

Fig. 3b

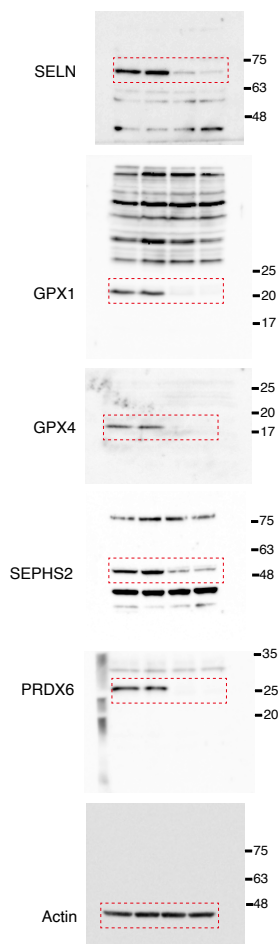

Fig. 3c

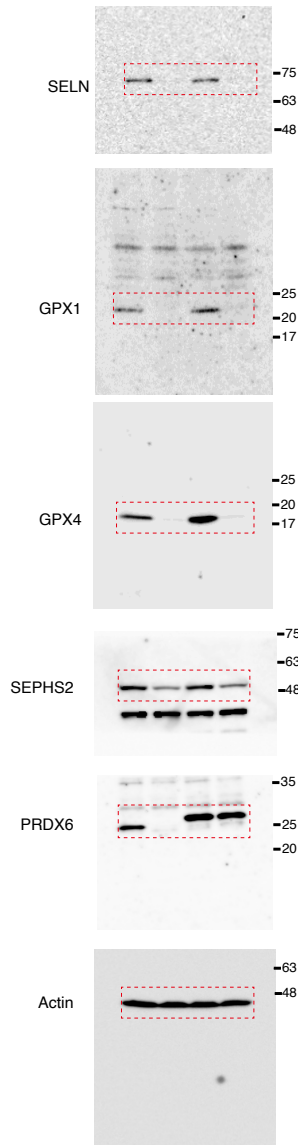

Fig. 3d

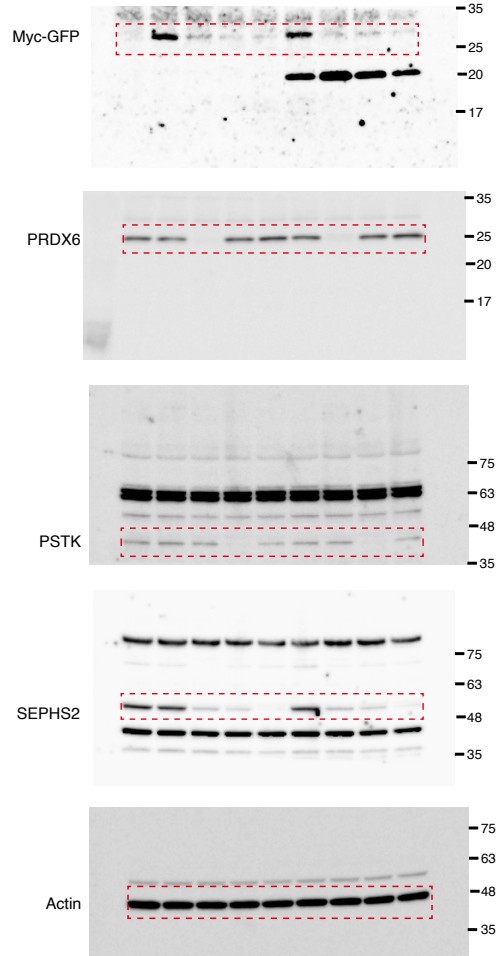

Supplement: Supplementary file 6 — Uncropped western blots [file 41594_2024_1329_MOESM6_ESM.pdf]

Fig. 4a

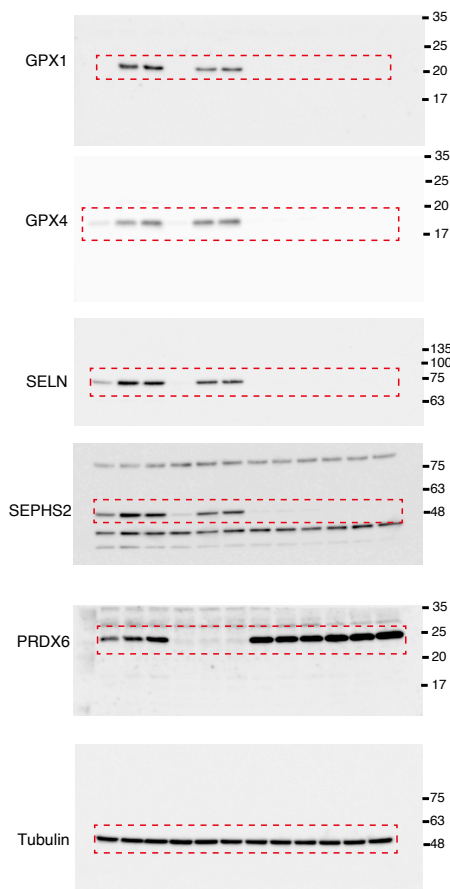

Fig. 4d

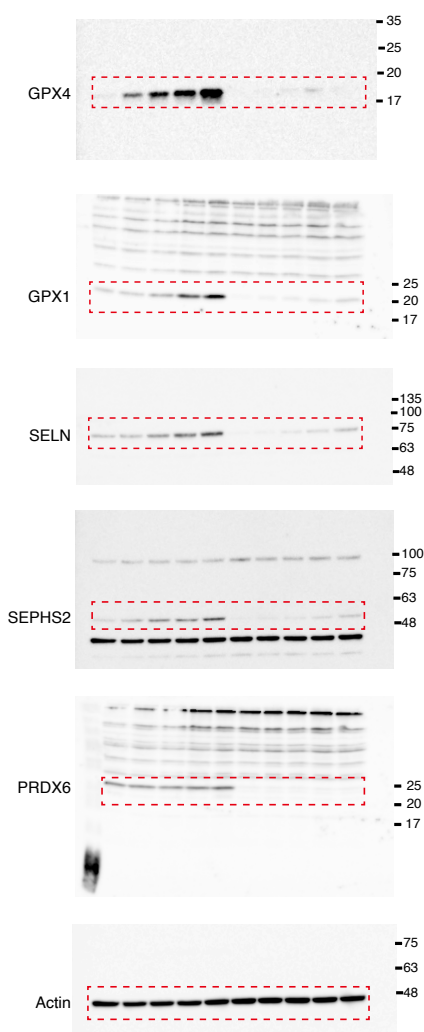

Fig. 4e

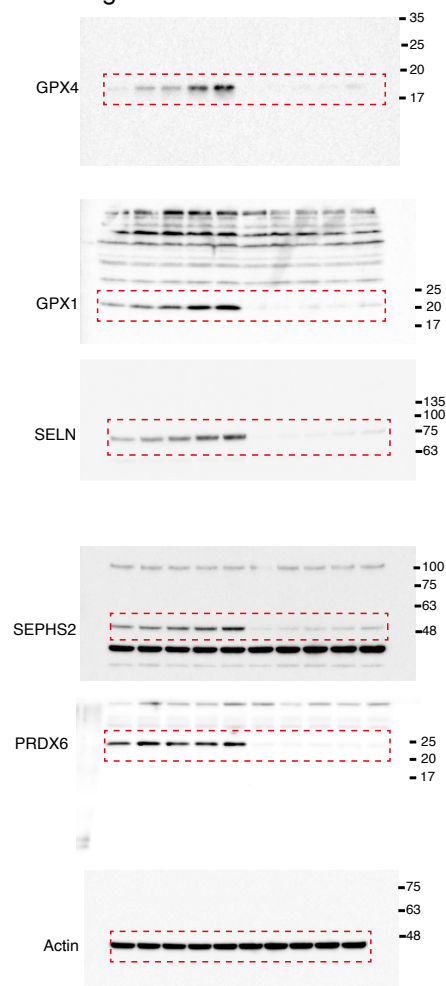

Fig. 4f

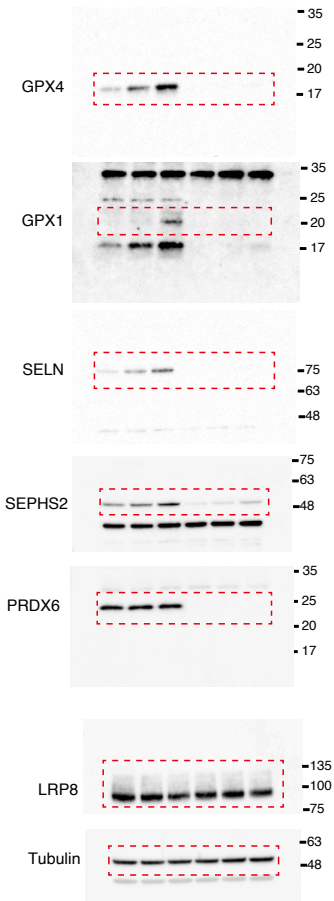

Supplement: Supplementary file 7 — Uncropped western blots [file 41594_2024_1329_MOESM7_ESM.pdf]

Fig. 5a

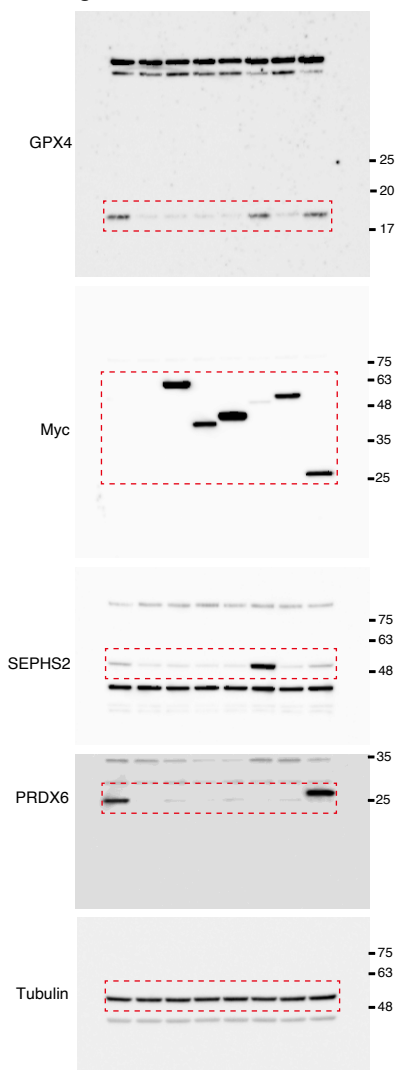

Fig. 5b

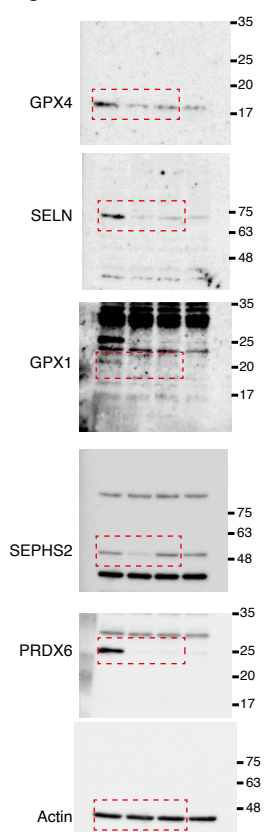

Fig. 5g

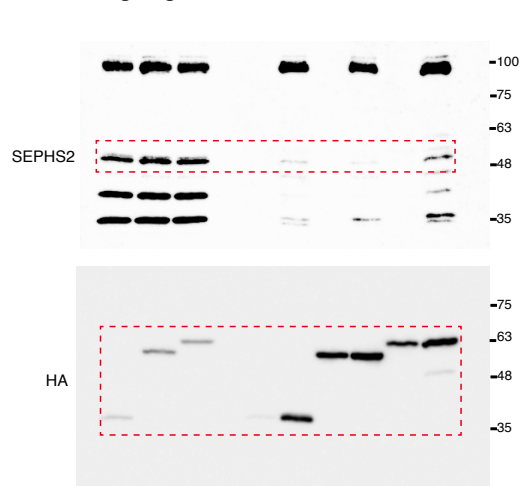

Supplement: Supplementary file 8 — Uncropped western blots [file 41594_2024_1329_MOESM8_ESM.pdf]

Extended Data Fig. 1d

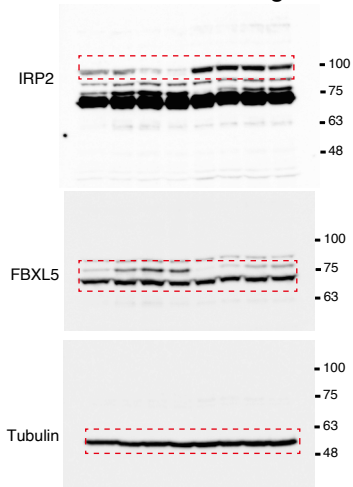

Extended Data Fig. 1f

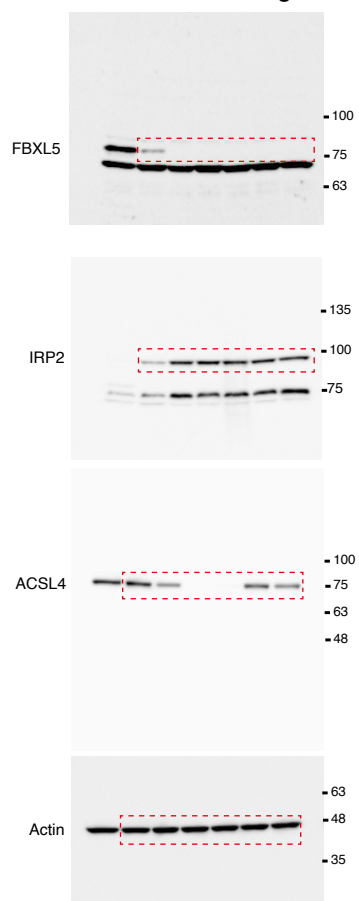

Extended Data Fig. 1g

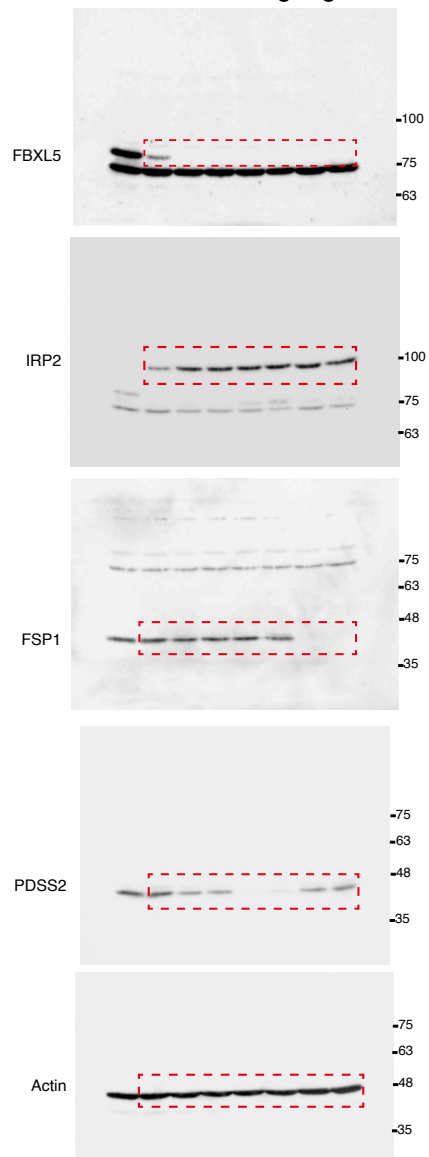

Supplement: Supplementary file 9 — Uncropped western blots [file 41594_2024_1329_MOESM9_ESM.pdf]

Extended Data Fig. 3a

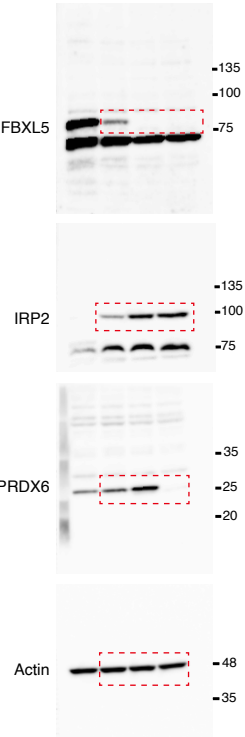

Extended Data Fig. 3b

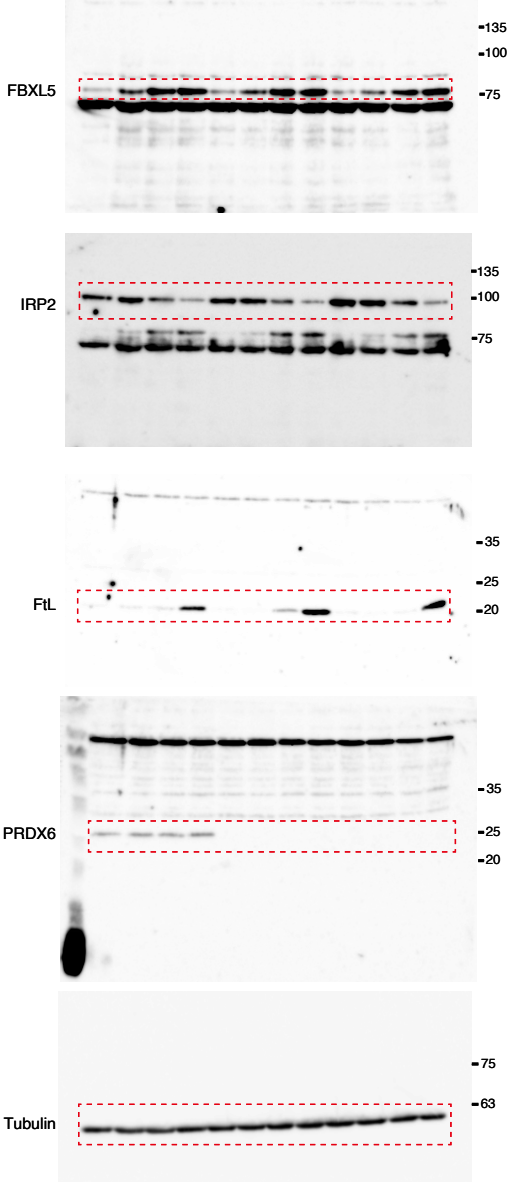

Extended Data Fig. 3d

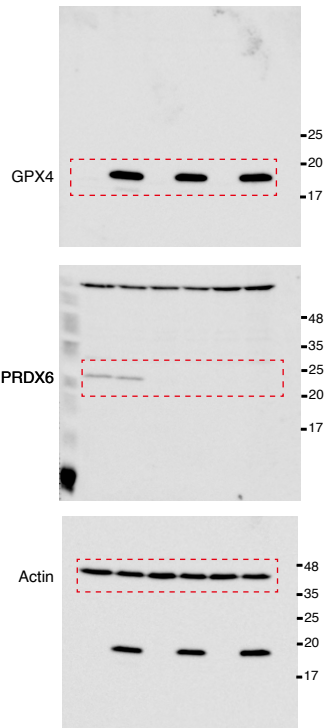

Extended Data Fig. 3i

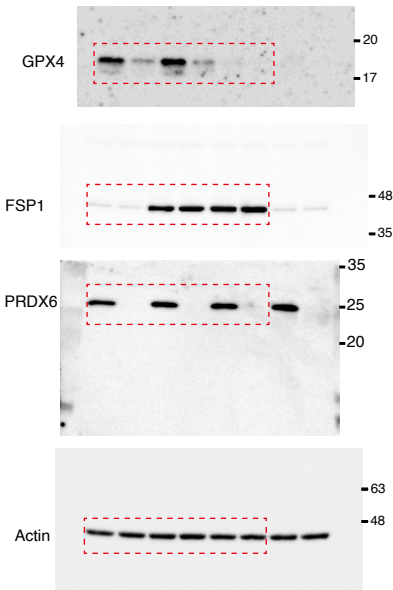

Supplement: Supplementary file 11 — Uncropped western blots [file 41594_2024_1329_MOESM11_ESM.pdf]

Extended Data Fig. 4b

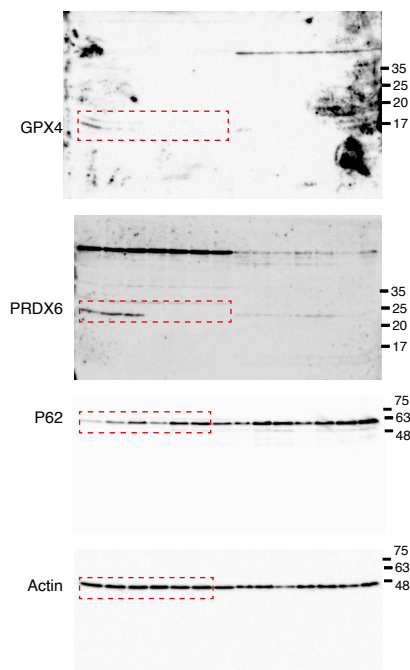

Extended Data Fig. 4c

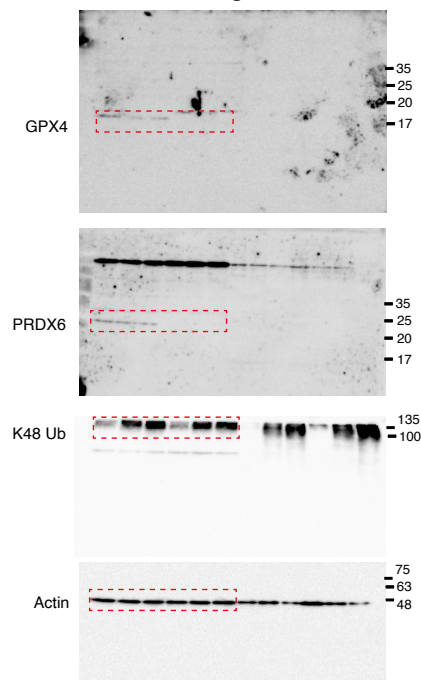

Extended Data Fig. 4d HeLa

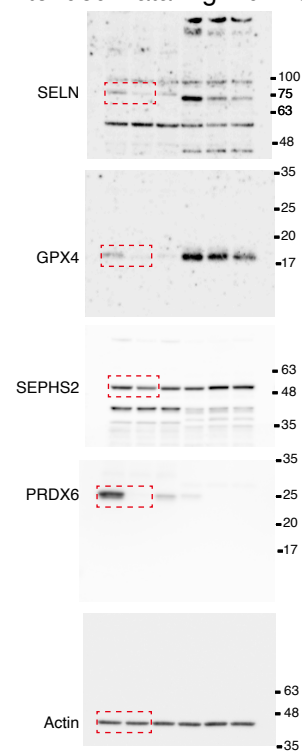

A549

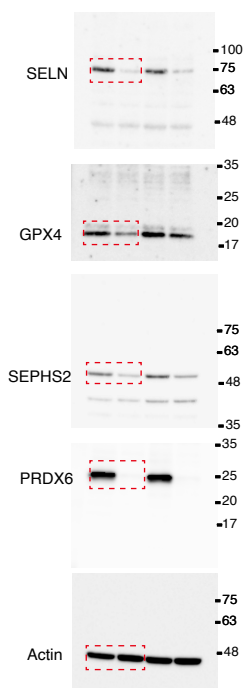

H226

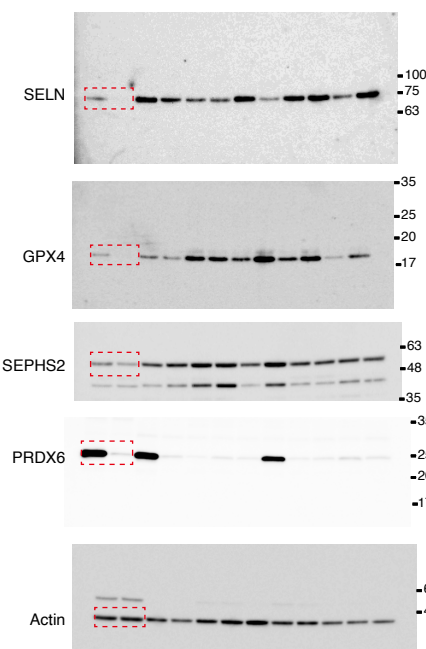

HepG2

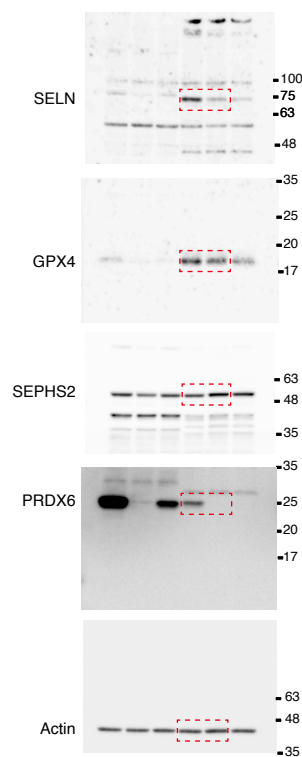

NB-1

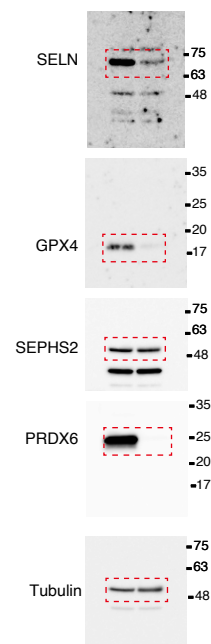

Extended Data Fig. 4e

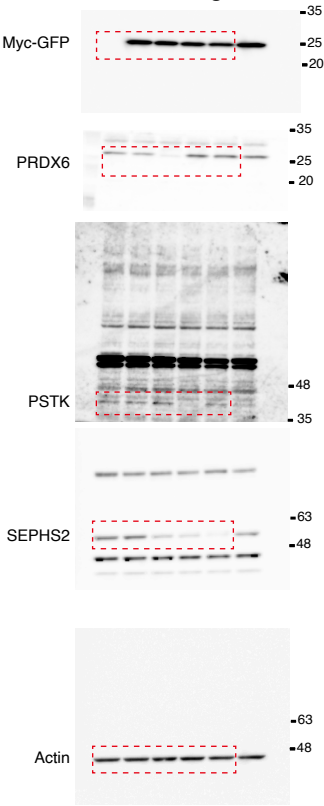

Supplement: Supplementary file 12 — Uncropped western blots [file 41594_2024_1329_MOESM12_ESM.pdf]

Extended data Fig. 6a

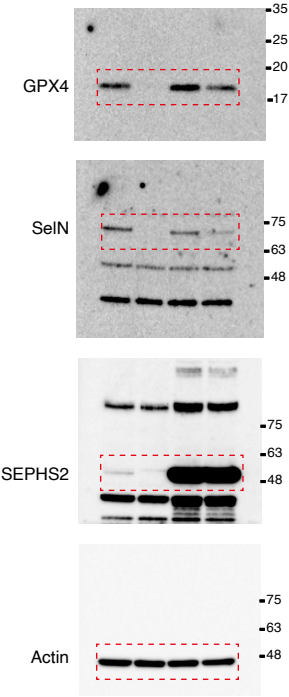

Extended data Fig. 6b

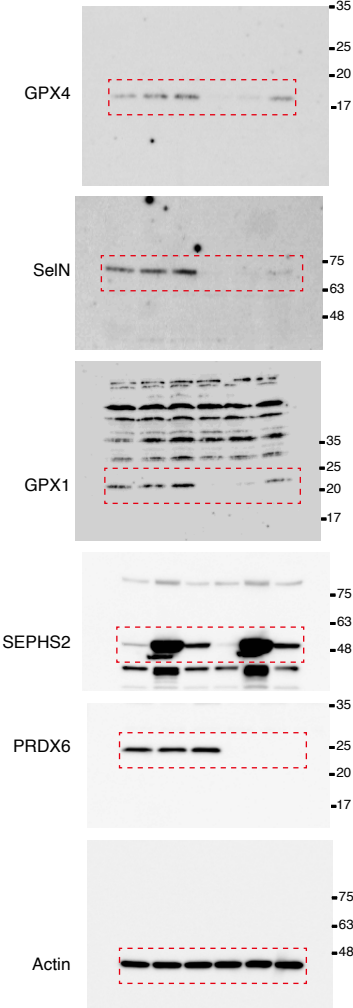

Extended data Fig. 6c

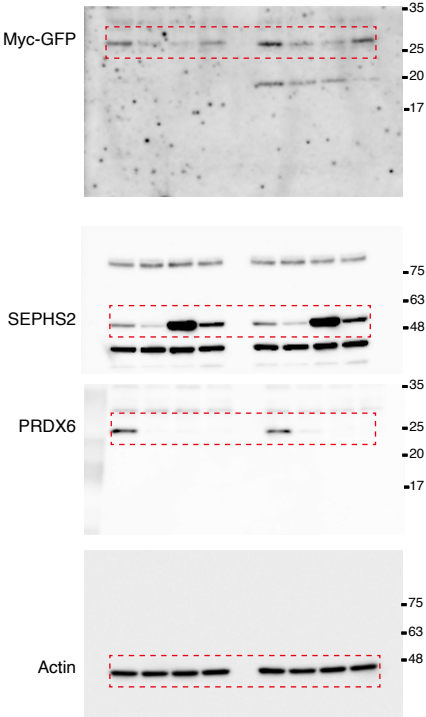

Extended data Fig. 6h

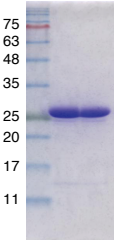

Supplement: Supplementary file 14 — Uncropped western blots [file 41594_2024_1329_MOESM14_ESM.pdf]

Extended data Fig. 8b

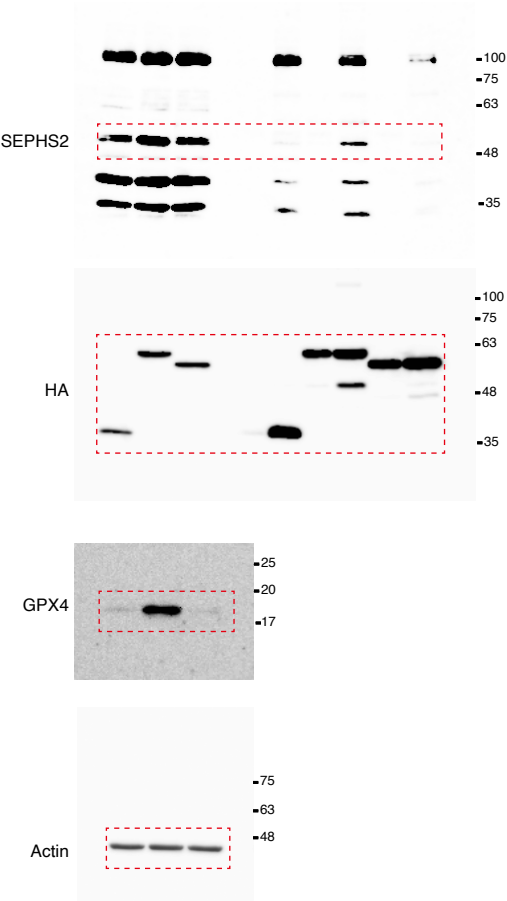

Extended data Fig. 8c

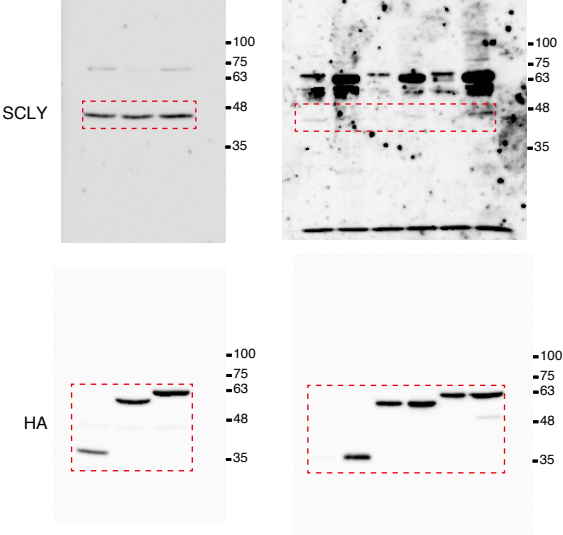

Supplement: Supplementary file 15 — Uncropped western blots [file 41594_2024_1329_MOESM15_ESM.pdf]

Extended data Fig. 10a

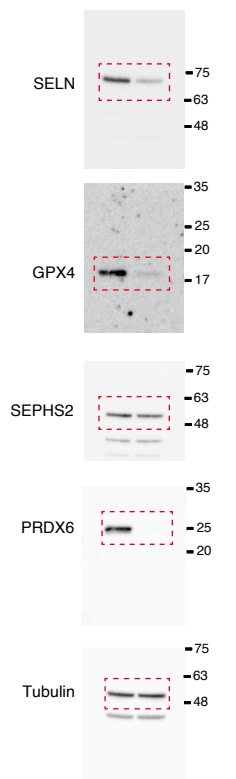

Extended data Fig. 10c

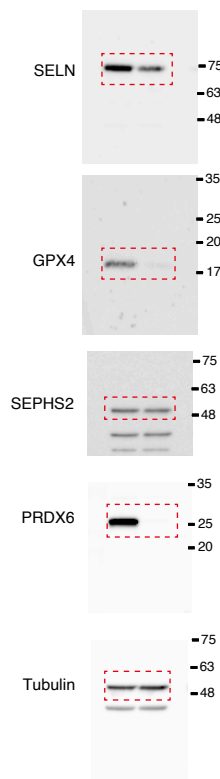

Extended data Fig. 10e

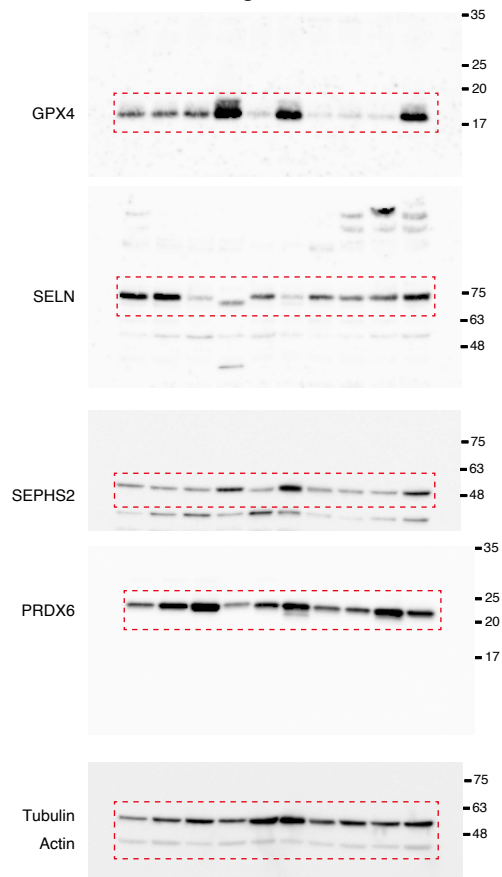

Extended data Fig. 10g

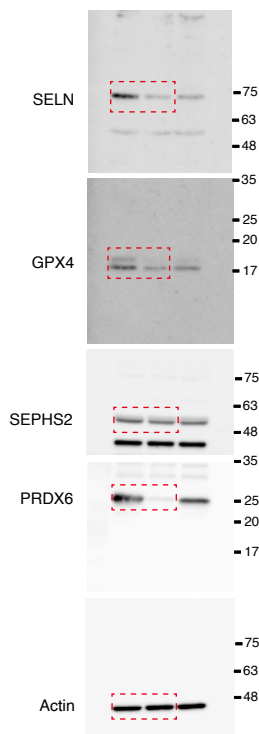

Supplement: Supplementary file 16 — Uncropped western blots [file 41594_2024_1329_MOESM16_ESM.pdf]
