# Supplementary material for: PRDX6 augments selenium utilization to limit iron toxicity and ferroptosis
Source: Nat Struct Mol Biol. 2024 Jun 12;31(8):1277–85. doi: 10.1038/s41594-024-01329-z (PMC11327102; doi:10.1038/s41594-024-01329-z)

Extended Data Fig. 2d

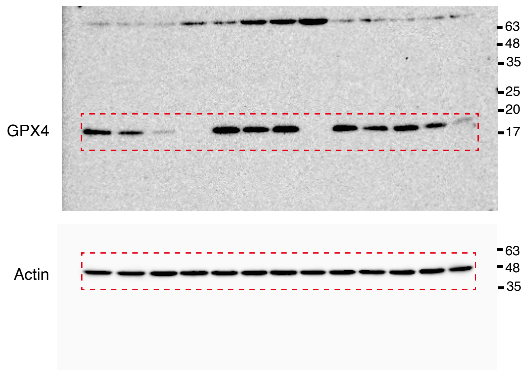

Supplement: Supplementary file 10 — Uncropped western blots [file 41594_2024_1329_MOESM10_ESM.pdf]
