# Supplementary material for: PRDX6 augments selenium utilization to limit iron toxicity and ferroptosis
Source: Nat Struct Mol Biol. 2024 Jun 12;31(8):1277–85. doi: 10.1038/s41594-024-01329-z (PMC11327102; doi:10.1038/s41594-024-01329-z)

Extended data Fig. 5d

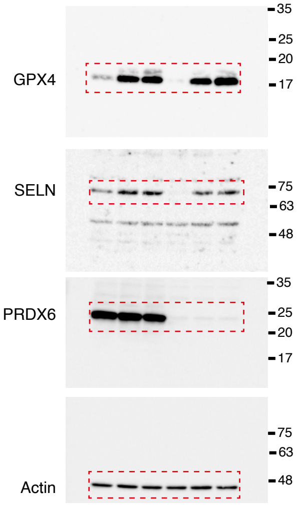

Extended data Fig. 5f

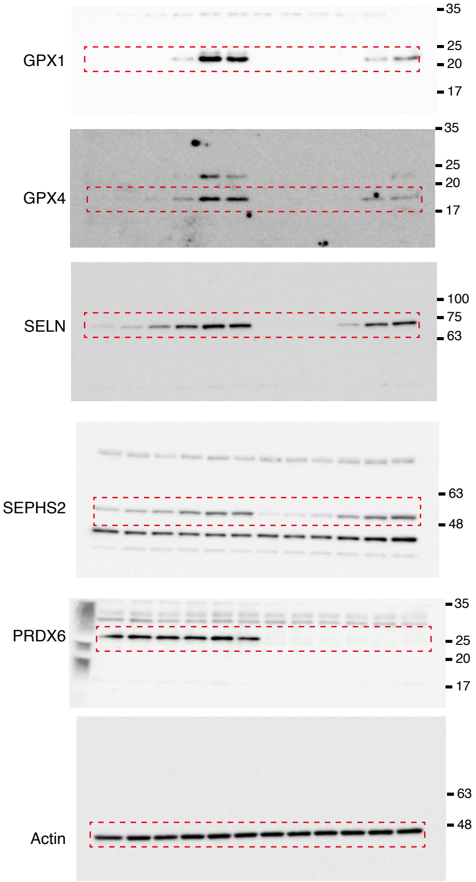

Extended data Fig. 5g

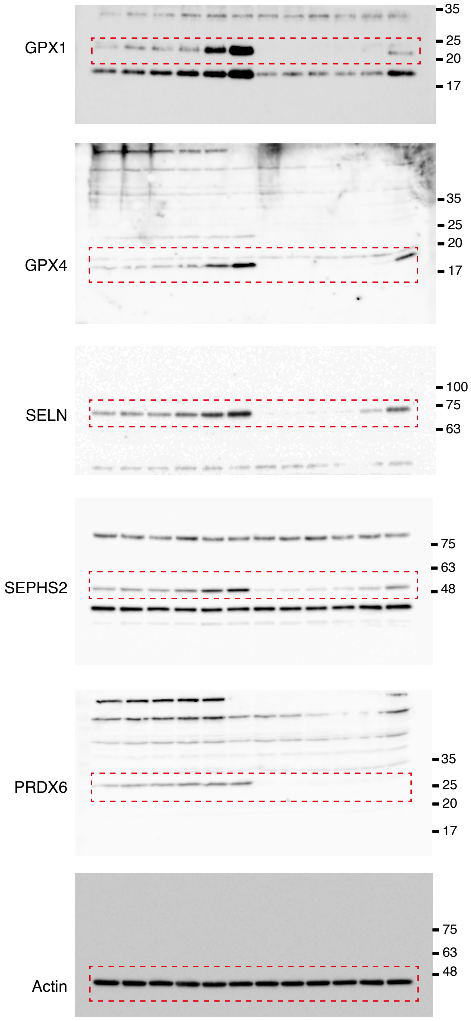

Extended data Fig. 5h

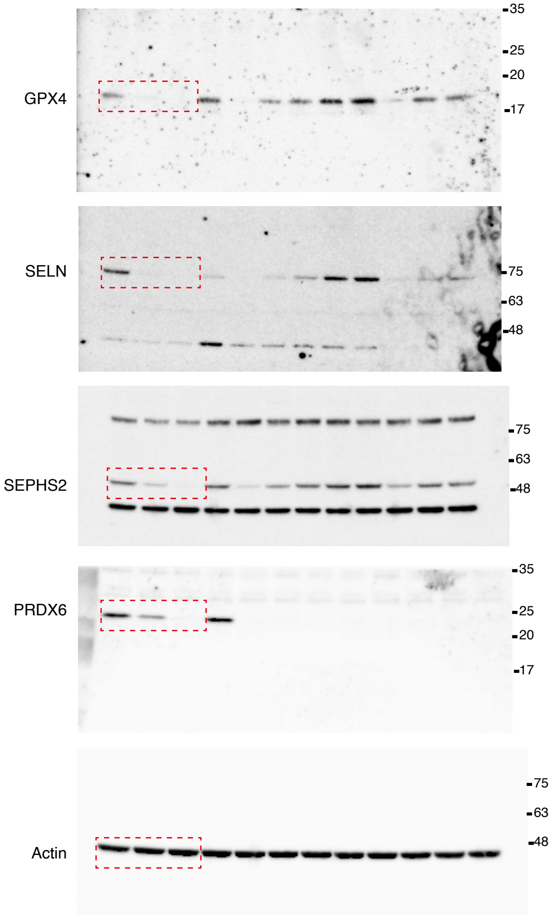

Extended data Fig. 5h

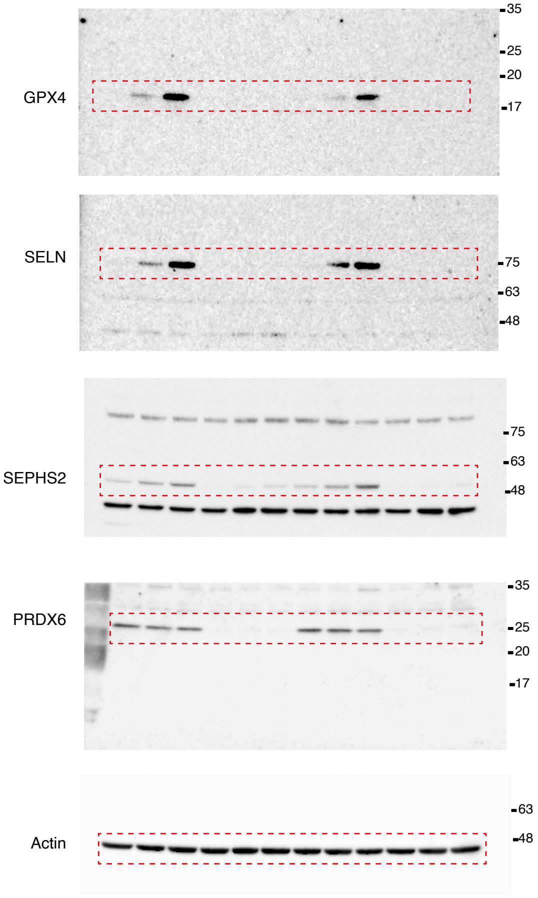

Extended data Fig. 5i

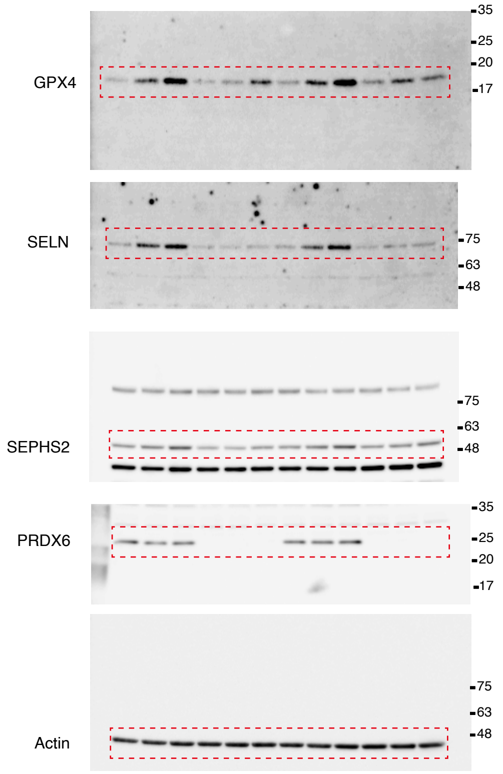

Supplement: Supplementary file 13 — Uncropped western blots [file 41594_2024_1329_MOESM13_ESM.pdf]
